# Book Reviews

**Published:** 1803-07-01

**Authors:** 


### CRITICAL ANALYSIS

OF THE

### RECENT PUBLICATIONS

ON THE

### DIFFERENT BRANCHES OF PHYSIC, SURGERY, AND MEDICAL PHILOSOPHY.

---

*The Natural History of the Human Teeth, including a particular Elucidation of the Changes which take Place during the second Dentition, and describing the proper Mode of Treatment to prevent Irregularities of the Teeth. To which is added, an Account of the Diseases which affect Children during the first Dentition. Illustrated with Thirteen Copper-plates. By* Joseph Fox, *Member of the Royal College of Surgeons, London; and of the Society of Medicine, Paris.* Quarto, pp. 113. London, 1803.

The author of this truly useful, accurate, and judicious work, after stating the great and increasing attention now paid to the teeth in country towns as well as the metropolis; the great increase of this branch of surgical practice, and consequently the necessity of practitioners duly qualifying themselves, observes, "A knowledge of the changes which the teeth undergo, and the circumstances which attend the progress of the second dentition, is highly necessary, as no safe or successful practice can be expected without a correct acquaintance with the anatomy and natural history of those parts.

"Mr. Hunter's work is the best book upon this subject in our language; but although he speaks of the second dentition, and of the irregularity of the teeth, it is only in general terms; neither has he given an exact or precise description of the proper treatment, so as to lead any person to undertake with confidence the right management of the teeth during this period.

"The extensive acquaintance I have had (says Mr. F.) with medical men, has given me an opportunity of discovering how useful and important they would deem a clear and practical work upon the different stages of dentition. Being possessed of a series of preparations, exhibiting the teeth under all their changes, and having been honoured with such a share of practice as to enable me to speak with confidence, I have ventured to present to the public those observations which I have been able to make, accompanied with engravings accurately illustrating the subject."

The author then proceeds to treat, in a concise and practical manner, 1. Of the Formation of the temporary Set of Teeth. 2. Of the permanent Set. 3. Of the Manner in which the Teeth are formed, formed. 4. Of the Shedding of the Teeth. 5. Of the Irregularity of the Teeth. 6. Of the Means of preventing it. 7. Of the Means of curing or remedying Irregularity. 8. Of supernumerary Teeth. 9. Of the Decay of the temporary Teeth. 10. Of the Diseases which attend Dentition. 11. The Analysis of the Teeth, by Mr. Pepys.

The book is beautifully printed, and the plates are particularly fine, and well chosen to illustrate the subject.

Every reader of this valuable work, we believe, will confess that the author has omitted nothing that can be of practical utility, nor inserted any thing that is not highly interesting to the physiologist as well as the practitioner.

---

*An Account of the Discovery of the Power of Mineral Acid Vapours, to destroy Contagion. By* JOHN JOHNSTONE, M.D. 8vo. pp. 38. London, 1803.

THE object of this pamphlet is to assert the priority of discovery in favour of the late Dr. James Johnstone, senior, of Worcester.

Dr. J. after a few observations on the use of acids internally in fevers, which have been recommended from the earliest times, proceeds to state the dates and facts respecting fumigation.

"In 1802, the Report of the Committee of the House of Commons, on Dr. C. Smyth's petition, states a sentence of Dr. Lind; 'That a certain method of destroying infection in places from whence persons cannot be removed, is a desideratum not yet obtained in physic. Many things had been proposed and tried, but without effect.'

"Nearly fifty years before the framing of this Report of the Committee of the House of Commons, a country physician had acquired eminence by the discovery of a certain method of destroying infection, which could be used, with perfect convenience, in the apartments of the sick. In 1758, Dr. James Johnstone published his 'Historical Dissertation concerning the malignant epidemical Fever of 1756, with some account of the malignant diseases prevailing since the year 1752, in Kidderminster.' In that dissertation, adopting the theory of the day, he proposes to keep the air free from putrefaction, by the steams of vinegar; or, as a more effectual method, 'the marine acid may be raised very easily, by putting a certain quantity of common salt into a vessel, kept heated upon a chaffing dish of coals; if to this a small quantity of oil of vitriol is, from time to time, added, the air will be filled with a thick white acid steam.' It is fortunate for the fame of Dr. Johnstone, that this discovery was published at the time. He had used the mineral acid vapour to correct the contagion of putrid fever in his earliest practice. The advantage derived from it became so well known in Kidderminster, that the manufacturers, during the prevalence of fevers in that town, spontaneously placed the fuming vessels in their shops; and Dr. J. continued to use the muriatic vapour in his extensive tensive practice, to the last hour of his life ; yet all this would have availed little, had it remained a mere matter of prescription. It would have been neglected or undervalued, and perhaps the practice and discovery altogether denied. I shall not dwell on these possibilities. It *was* published in 1758, as having been practised in 1756; and the book attracted so much notice, that the whole edition was quickly sold.

" But it falls to my lot to discharge an important duty to science, and to the memory of my father. It is denied, that the muriatic vapour can be used with convenience in the apartments of the sick; and some men affect to doubt, whether it be of equal efficacy with the nitric acid vapour, which has been lately (near fifty years after the muriatic was used for the same purpose) called a discovery. On both these points I shall appeal to a few simple facts.

" It would be useless to enter upon such a detail of cases as I could collect from the books of my father, consisting merely of names and prescriptions, without any regular statement of symptoms, or of the particular operation of medicines. Such a detail would only prove that the muriatic vapour was used subsequent to the year 1756, of which any proof is unnecessary, since the publication of the Dissertation on Fevers.

" Mr. Crane, the present eminent and respectable surgeon in Kidderminster, is prepared to give the fullest testimony, that the muriatic acid vapour was so commonly used, when he first settled in business, more than thirty years ago ; that the manufacturers placed it spontaneously in their shops, when fever was apprehended ; and that, in malignant cases, it was always ordered by my father. He remembers Mr. Cooper and Mr. Symonds, two old and eminent surgeons of Kidderminster, who were employed with my father in his earliest practice, to have frequently mentioned the discovery of the muriatic vapour, and the use of it, in the fever of 1756 ; and its continual use, when occasion called for it, from that period.

" Dr. Johnstone's method of using muriatic acid vapour was as follows:—Put one pound of common salt into an earthen vessel, and pour over it, from time to time, a small quantity of vitriolic acid, till the whole salt is moistened. If the air is foul, and peculiarly offensive, the vessel may be heated, to extricate a larger quantity of vapour ; but, in a common way, the addition of the acid to the salt will answer, without applying heat, in rooms from fifteen to twenty feet square.

" Dr. Smyth's formula is :—Put half an ounce of vitrolic acid into a crucible, or glass, or china cup ; warm this over a lamp, or in heated sand, adding to it, from time to time, some nitre. These vessels should be placed at the distance of twenty or thirty feet from each other, according to the height of the ceiling, or virulence of the contagion.

" If, in Dr. Smyth's way, only such a quantity of nitre be added, from time to time, to ½ an ounce of heated vitriolic acid, as shall be decomposed decomposed gradually in the space of 15 square feet, no wonder that the vapour should be productive of little inconvenience, for it must be in so diluted a state as to be scarcely perceptible. In the same space, as much common salt as can be decomposed by two ounces of vitriolic acid may be borne without any cough, or interruption of respiration, if the immediate stream of rising undiluted vapour be avoided, i. e. four times as much. According to Dr. Smyth's directions, an apparatus, and the application of heat, are required, which are always troublesome in a sick room.

"Dr. Johnstone's whole apparatus is an earthen dish filled with salt, which may be placed in a corner, and requires no attention.

"The muriatic vapour is always attended by a visible cloudiness of the air of the apartment, a quantity of humidity being precipitated as it rises.

"The nitric vapour is more transparent and invisible, and in one instance suffocation had nearly taken place in a very large room, from its over accumulation. I do not deny that muriatic vapour may also suffocate, or excite cough, but it does not occasion these symptoms in a greater degree than the nitric. Nor do I deny that the nitric vapour may be used with efficacy and convenience to correct contagion. But I aver that the muriatic is equally efficacious, and may be used, at least, with equal convenience; that it is equally mild, and equally safe, in like quantities.

"The following facts will prove this assertion:"

Dr. J. then adduces a number facts and experiments to demonstrate the truth of his conclusions, and which appear to us highly satisfactory.

About a month ago, we received an anonymous communication from Staffordshire, in which the discovery of acid fumigation is given to the late Dr. James Johnstone, senior, of Worcester; and a case is detailed where it was successfully employed in arresting the contagion of the scarlet fever and sore throat.

---

*On the Influenza, as it prevailed in Bristol and its Vicinity, during Part of February, March, and Part of April,* 1803. *By* John Nott, M. D. Octavo, pp. 25. Bristol, 1803.

"The history of epidemics," says Dr. N. "is perhaps the most curious and interesting of all researches in medicine and philosophy; no diseases are involved in more obscurity; none have received less light from investigation; and, could we satisfactorily trace their origin, none would give us greater insight into natural causes; but such discussion does not belong to these pages."

After mentioning the similar epidemics recorded by authors from 1510 to this time, Dr. N. proceeds to consider the *remote* causes, and thinks them ascribable to some peculiarities in the constitution of the air, which have hitherto eluded all medical research.

"The *predisposing* causes must rest with the persons attacked by it: those, who from an irritable state of the Schneiderian membrane, brane, or from extreme tenderness and susceptibility of the lungs, are most liable to the common catarrhal cold, have been most readily affected.

"The *proximate* causes, I presume, are those morbific miasmata, of whatever nature they may be primarily, which float in the air, and, being inhaled by the mouth and nostrils, excite the disease.

"How far the halitus, and contact of those infected with it, can be immediate causes, involves the question of contagion, which has been much controverted; it has found, for and against it, many and zealous partisans; I am not competent to decide upon it; but I have strong reasons to entertain doubts of the disorder being contagious.

"I have seen two or three persons of a numerous family attacked with this influenza, and after a fortnight, perhaps, they have completely recovered. Another fortnight has elapsed, and others, who have sat at meals at the same table, been constantly in the same society with those first infected, have then fallen ill; the first infected not partaking of the complaint a second time. Surely this is very unlike contagion. If contagious, when any individual of a society had the disease, it is most likely the rest of the same society would have caught the infection one after another; and those first infected would have had it a second time, on the rest falling ill, who might, in the first instance, by accident, have escaped; for it does not seem a law of the disease to attack but once: many have relapsed, who, to all appearance, were got perfectly well.

"Again, I have known whole families, considerable in number, affected with it, masters and servants, on one and the same morning, or evening perhaps; to suppose that each caught it from the other, argues a rapidity of infection, that no poison hitherto known, I fancy, ever yet occasioned. But these persons were living in the same atmosphere, under the same circumstances, most likely on the same diet, and from similar habits acquiring, possibly, similar dispositions towards susceptiblity. This may account for such a sudden general attack.

"Numerous instances also have happened within every person's knowledge, of married couples, one of whom has contracted the complaint, while the other has continued exempt during the whole period of its prevalence; without the idea of separate beds once occurring, or being adopted as a prevention.

"Nor does any marked boundary seem to restrain its predominance. Surely the width of the British Channel, and the adjoining extents of land on either coast, were sufficient to prevent contagion passing between London and Paris; at both which places it raged with violence at the same exact period. Indeed, in former influenzas prevailing here, foreign countries have never failed to be affected by them, in some quarter or other, though not so immediately at the same juncture.

"The great uncertainty, and sometimes rarity of its propagation in in public seminaries (which must be considered as forming a very excellent test) might, from the reports of well-informed and observant practitioners, incline us to an opinion of the malady not being contagious.

"Moreover, remote villages, and solitary houses, we learn from good authority, have been infected with it, at the very precise time it has appeared among large cities, where it would naturally make a more seeming rapid progress, from the predisposing causes prevailing more in them, and the general constitution of the animal frame being more weak and susceptible.

"My belief then is, till further investigation shall correct my judgment, that this influenza is epidemic only, not contagious."

On the treatment at the commencement, Dr. N. has made an observation which we believe is peculiar to himself.

"As this complaint is particularly sudden in its attack, so does it seem to admit of very sudden suspension. A full dose of tincture of opium, that is, perhaps thirty drops, taken at bed-time. has seemingly cured the patient for the whole of the next day; more especially, I have remarked, if any way suffering from its subsequent effects as a narcotic; but the succeeding day it has returned with redoubled violence, as though determined to have its course. From 30 to 40 drops of the following compound tincture, viz. tincture of fox-glove, tincture of opium, of each two parts, tincture of squill one part, mixed, taken once in six hours, would alike cause a suspension temporary only.

"To remedy this disorder, the same intentions of CURE as we observe in the common catarrhal cold were all that, I believe, our medical practitioners adopted. To equalise perspiration throughout the system, and for a time to keep it increased, seeing that all the other natural functions of the body were duly regulated, appeared the main object. In the influenza of 1782, some writers on the subject told us, that the disease passed off in proportion as a tendency to perspiration was observable; this shows how Nature would effect a cure of her own accord, and how nearly therefore we ought to imitate her in our intentions."

---

*Cases of the successful Practice of Vesicæ Lotura for the Cure of diseased Bladders. Part I. Second Edition, 8vo. pp. 47. Part II. with a Plate of the Apparatus: And also, Cases of diseased Affection from Phymosis, with a new Mode of operating, and Plate of the Instrument for performing it. By* JESSE FOOT, Esq. Octavo, pp. 92. London, 1803.

THESE pamphlets contain thirteen cases of diseases of the bladder, attended with great distress to the patients, cured, or greatly relieved, by injecting the bladder with proper fluids. To these the author has added six cases where the dysuria, and affections of the bladder and kidnies, proceeded from phymosis, or too small a perforation of the glans penis.

This

This work merits the attention of Surgeons, and is likely to lead to considerable improvements in the manner of treating diseases of the urinary organs.

---

*Medical Directions for the Use of Navigators and Settlers in Hot Climates. By* THO. M. WINTERBOTTOM, M. D. *Physician to the Colony at Sierra Leone.* 1803, pp. 144. 8vo.

AN intelligent medical man, who has resided four years at Sierra Leone, must be familiar with most of the diseases and accidents of tropical climates, with which Europeans are likely to be affected. The Author of this useful little work, introduces it to public notice in the following simple and unassuming manner.

"The medical directions contained in the following pages were originally written at Freetown on the coast of Africa, for the use of such trading vessels in the service of the Sierra Leone Company as were unprovided with surgeons.

"Having been favourably received, and applied, on many occasions, with a beneficial effect, they are now presented to the public under an enlarged form, with the hope that they may become more extensively useful to Europeans, whose pursuits, in hot climates, often lead them into situations where medical assistance cannot be obtained.

"The directions are purposely written in a plain, familiar manner: in order to render them more intelligible, and more easily applicable to practice, a few cases have been added of persons labouring under some of the diseases described. Technical terms have been carefully avoided, and no expressions are used, but such as, it is presumed, must be well understood by the generality of readers.

"The methods of cure recommended, are for the most part such as the author found successful during his residence at Sierra Leone, where, for nearly four years, the health of more than fourteen hundred persons was entrusted to his care."

The first observation which the author makes is rather too general. He says, "Europeans upon their first entrance into a tropical country are too apt to have their minds impressed with ideas of danger from the climate. The great increase of heat is the first inconvenience they experience, and in general, that which they most dread, &c." It is true, that the risk to health incurred by a change from cold or temperate to tropical climates, is a matter of common observation, and is a great source of anxiety to the friends of those whose duties or pursuits lead them thither: but has not melancholy experience shewn, in numerous instances, this apprehension is but too well founded? The fault of Europeans, at least of Englishmen, in such circumstances, is too blind a confidence in the vigour of their constitution, and an obstinate rejection of those precautions which the experience even of the natives of tropical climates (the best judges in these instances) has uniformly shewn to be requisite. If the utmost difficulty is found in prevailing upon

 the the Englishman, first entering into tropical climates, to moderate his intemperate practices, to give up some of his European habits, to use the precautionary measures of due clothing, avoiding damps and the like, it can hardly be maintained that he is likely to err from excess of apprehension of disease.

Some very proper directions for diet and general management of health immediately follow: they are such as have been inculcated by Lind, Pringle, Huxham, and many others; but we do not recollect having ever seen them drawn up with more good sense and unaffected plainness. The author is a great advocate for flannel clothing. He says, "The clothing ought to be light, but sufficiently warm to guard against sudden changes from heat to cold. Cotton and flannel are the best articles for dress. Short jackets, with trowsers reaching to the ancles, and fastened by strings or buttons, are most convenient; where stockings are not worn, socks should be used in their stead: the practice of going without shoes is highly improper. Hats with high crowns, or with a handkerchief put in them, are of great service in protecting the head from a hot sun. The use of flannel next the skin cannot be too strongly recommended; for although the first wearing of it may produce some trifling inconveniences, such as, an itching in the skin, and an increased eruption of the prickly heat, yet these seldom last longer than a few days. By constant use, the wearing of flannel becomes remarkably pleasant, and to it many persons are indebted for the preservation of their health and lives in very hot climates. Being defended with flannel, they have suffered no inconvenience, even when exposed to the tropical rains for whole days and nights in open boats, or in the woods; while others, neglecting to use the same precaution, were presently after seized with fevers. During the rainy season, the air often feels raw and chilly; and the changes from heat to cold are more sudden than at other times: but although the good effects of flannel are then more apparent, it is nevertheless extremely beneficial in the dry season also; after violent perspiration, a linen or cotton shirt becomes wet and always cold to the back, which never happens when flannel is worn. Any considerable alteration of the temperature of the air requires a proportionate change in the dress. Those especially who are exposed to the night air should put on additional clothing. In the rainy season, woollen cloaths will be found both comfortable and necessary. It is a very frequent custom with those who have resided long in Africa, to wear only a shirt and trowsers, and in this dress to expose themselves at night when the land breeze blows; or at other times to sit in a current of air: but, however agreeable the present gratification may be, it is always followed by a feverish dry skin, owing to a stoppage of perspiration, and very often proves a cause of violent diseases; so that it is a practice by no means to be imitated by new settlers.'

The inconveniences and accidents of ship-board are next considered; such as sea-sickness, and the best means of guarding against, or or shortening the period of that distressing event; scurvy, and other circumstances of importance.

The author next proceeds to describe the disorders that occured at Sierra Leone, and which occur in all similar climates. The first and most important class of diseases is fever; its course is known by fatal experience to be dreadfully rapid in these climates, and hence its first obscure symptoms should be watched with anxious care. The author divides the fever of hot climates into the two species of remittent, or what is usually termed emphatically *the fever*; and intermittent, or ague. For the present purpose this distinction may be sufficient. Ague is first described: its symptoms must be familiar to all our readers; we shall therefore only mention the circumstances that apply peculiarly to hot climates. Here the author remarks: "Much mischief has arisen in consequence of an opinion which is too generally entertained, that whenever a cold fit takes place, no danger is to be apprehended; and the patient's friends are confirmed in this opinion, as they observe that after lying down a few hours while the fit is upon him, he has frequently a keen appetite as soon as it is over, and can return to his work as usual. The fits likewise, after several returns, seem to become milder; and sometimes wholly cease for a few days, a week, or a month, and then return only for a short time: this deceives the patient, who thinks the disease is gradually going off, while, on the contrary, it grows more inveterate, and is undermining his constitution. It cannot be too often repeated, that we ought never to neglect, nor think slightly of an ague: by not attending to this maxim, many Europeans on the coast of Africa have been irretrievably injured; for experience sufficiently proves, that the consequences of a neglected or mismanaged ague are, in tropical climates, more fatal than even the effects of a continued fever."

The cure is to be effected by the bark. A simple case of ague is added in illustration; an useful plan, which the author pursues throughout.

He then proceeds to describe the remittent fever, a more dangerous complaint than the former, and the most fatal disease affecting Europeans on the coast of Africa. The following are its symptoms: "The manner in which the fever comes on is different in different people; it frequently appears as a common cold, producing slight head-ache, chilliness, and wandering pains over the body; but is so slight that the patient scarcely thinks himself unwell. With these deceitful symptoms it continues three or four days, until the great weakness of the patient, or some sudden change in the disease, raises an alarm. It is thus, probably, that many persons are said to be carried off by fevers within forty-eight hours: whereas, had the time been reckoned from the first sensation of illness, it would have been six or seven days, or more.

"The patient sometimes feels uneasy for a day or two before any febrile symptoms take place; there is a heat and heaviness of the eye-lids, so that he can scarcely keep them open; or a burning heat in in the palms of the hands and soles of the feet; he feels the skin dry, rough, and uncomfortable; complains of a loss of appetite and bad taste of the mouth; and has a sensation of anxiety, or universal oppression, not easy to be described. The previous sensations, however, are so numerous and varied, that almost every person who has had the disease, is conscious of its future attacks by a set of feelings peculiar to himself. There is sometimes a dark sallow complexion, with a heavy dull eye, and a want of activity, which continue for a day or two, and point out the approach of fever to a by-stander, though the patient makes no complaint himself.

"The fever usually commences with shiverings, not very severe, and sometimes scarcely noticed by the patient. The shivering is succeeded by the symptoms which occur in the hot fit of an ague; but the restlessness and anxiety are in fever generally greater: the pain of the head also is more distressing, and seems often as if produced by a sharp instrument fixed over the eyes: at other times there is a sense of tightness over the forehead, as if the head were bound round with a cord. There is a burning heat in the skin, though to the patient it feels rather cool, which is apt to mislead; but if the hand at this time be applied over the forehead, the heat will be found very great. The patient tosses about, and seeks ease in every part of the bed: sometimes however he lies upon his back, inwardly moaning, without making any specific complaint. In this case, if the hand be applied over the stomach so as to press it gently, the uneasiness is in general increased. There is seldom much delirium, but the patient seems confused, and gives very quick answers to the questions put to him: if he fall into a sleep it is for the most part uneasy, and interrupted by frequent startings.

"When these symptoms have continued with more or less severity for a few hours, a sweat breaks out, first about the neck and breast, sometimes, though seldom, spreading over the whole body: this sweat is more likely to be diffused over the body after the first fit than after any of the succeeding ones. The relief which follows it after the first fit is sometimes so great that the patient thinks himself well, though the head-ache does not go off so entirely as in the ague; and his strength remains greatly depressed. This relief of the symptoms, or remission, lasts only six or eight hours, when another fit comes on, generally a much more severe one. The second paroxysm being seldom preceded by a cold stage, seems to consist only of a hot fit, more especially as the sweating stage, which should follow it, does not appear with any regularity at the second or third return of the paroxysm. In short, all the symptoms become more violent, and the remission less perceptible every succeeding fit. The fever goes on in this manner until the patient's strength is entirely exhausted: a fatal termination seldom happens before the fifth or sixth day, but takes place generally about the eleventh or twelfth day, if the complaint has been neglected in the beginning."

The plan of cure laid down is judicious, and agrees in many particulars with that of the most respectable authorities. The author next describes the symptoms of the yellow fever, principally by way of diagnosis from the last mentioned. It does not appear whether he writes in this case from experience, though the example of the too celebrated Boulam fever, shews that the virulence of this disease is not confined to America or the West Indies.

"The yellow fever," he observes, "mostly affects persons just arrived from Europe, or from the northern parts of America; particularly those of a robust, full habit of body, who appear in the highest health. It is most prevalent during the hottest months of the year, and is often a consequence of too violent or too long continued exercise in the sun. It is more frequent on the sea-coast, and in a heated dry sandy soil, than in the interior parts of the country: and in mountainous situations it rarely occurs. The remittent fever, on the contrary, appears chiefly in low swampy situations, in uncultivated places, and stagnant air; and it chiefly attacks such as have resided some time in the West Indies, especially when weakened by previous illness, or of a delicate, relaxed habit of body. The yellow fever attacks very unexpectedly, and with very great violence, while the patient thinks himself in the most vigorous state of health." He then enumerates the symptoms, which we shall not transcribe, as they agree with the description given by Rush and other authorities. Dysentery does not seem to be very frequent at Sierra Leone. Cholera Morbus, being chiefly occasioned by sudden transition from heat to cold, appears not to be unfrequent here; it is described with great clearness. We were rather surprized to find that Tetanus had not been known at this colony; and the Author adds, we doubt whether quite correctly, that "it appears to be scarcely known upon the coast of Africa." It is however well described. Insolation, or the *Coup de Soleil*, is equally unknown in Sierra Leone: but there occurs frequent fulness and throbbing of the temples to Europeans who expose themselves much to the sun.

Several other diseases of the most usual occurrence, both in hot climates and in general among sailors, are added, such as rheumatism, rupture, small-pox, lues, ulcers, wounds, &c. The description is such as can be readily understood; and the mode of practice, generally, can be easily followed. A short Appendix is added, containing a select list of medicines for the coast of Africa, with plain directions for their use. The list of medicines we shall give: the quantity of each is stated to be sufficient for twenty men for one year.

Powder of Peruvian bark, ℔ x. Powder of Angustura bark, ℔ iv. Powder of Colombo, ℔ j. Powder of Rhubarb, ℥ x. Powder of Ipecacuanha, ℔ j. Tartar emetic, ℥ ii. Antimonial wine, ℥ viii. Opium, ℔ ſs. Laudanum, ℔ j. Calomel, ℥ xii. Sweet spirit of nitre, ℔ j. Oil of mint, ℥ iij. Huxham's tincture of bark, ℥ xii. Spirit of lavender, ℔ j. Epsom salts, ℔ x.

 Camphor,

Camphor, ℥ xii. Gentian root, ℔ vi. Cream of tartar, ℔ iv, Castor oil, ℔ vi. Gum Arabic, ℔ iii. Bilious or purging pills, No. 340. Magnesia, ℔ ij. Salt of tartar, ℔ j. Diluted vitriolic acid, ℔ j. Diluted nitrous acid, ℔ ſs.

Cerate, ℔ iij. Simple ointment, ℔ iii. Red precipitate, ℥ vi. Blue vitriol, ℥ ij. Blister plaster, ℔ iii. Powder of Spanish flies, ℥ iv. Extract of lead, ℔ ij. Sugar of lead, ℔ j. Spirit of hartshorn, ℔ ij. Flowers of sulphur, ℔ x. Sticking plaster spread on cloth. Tow, lint, rags, &c.

Stores for the Sick. Barley, ℔ xij. Sago, ℔ x. Tapioca, ℔ x. Portable soup, ℔ vi. Vinegar, ℔ viij. Concrete acid of lemon, ℔ ij.

To the above articles should be added, sugar, tea, coffee, molasses, onions.

The general execution of this little work is very respectable, the style is plain, unaffected, and so free from technical terms as to be readily understood by captains of ships, or any intelligent persons not of the profession. The impression of the whole is certainly to lessen the supposed danger to Europeans from tropical climates, a circumstance which at least, will give a favourable idea of the healthiness of Sierra Leone, in which settlement the Author's observations seem principally to have been made.

---

*Illustrations of some of the Injuries to which the Lower Limbs are exposed. By* CHARLES BRANDON TRYE, *Member of the late Corporation of Surgeons in London, and Surgeon to the Gloucester Infirmary.* pp. 37, 4to. with plates.

THE chief circumstance which gave rise to the publication of this work, the author states to be the rare opportunity which fell in his way of dissecting two cases, one of dislocation of the femur, the other of fracture of its articulating neck, within a short time after the accidents; an opportunity which certainly does not often happen, since these misfortunes are not of themselves attended with much risk to life.) Besides, the author, in his situation as surgeon to a very respectable Infirmary, must have been in the habit of observing many cases of dislocation of the limbs, and the present work shews that he has bestowed much attention on this important subject

The three first plates represent the dislocation of the femur, which was upwards and outwards, the articulating head of the bone being driven under the glutæus major, and by dissection was found to have formed, during the interval of twenty-two days between the accident and the death of the patient, a new ligamentous attachment to the remains of the ligamentum teres. On account of other injuries no attempt was made to reduce the dislocation. The fifth, sixth, and seventh plates are dissections of a case of fracture of the neck of the thigh bone, one of the most uniformly hopeless cases in surgery that ever occurs. The patient, an aged female, died of a bowel complaint six weeks after the accident. On examination, the capsular ligament was found entire and sound, the fractured parts were were united, but not firmly, by a great luxuriance of callus, the neck of the bone was twisted, the great trochanter being thrown backwards so as to advance the neck of the bone, and to force the little trochanter very near to the head of the bone. The author adds his testimony to that of several other eminent surgeons in the opinion, that no relief can be obtained in the calamitous accident of fracture of the neck of the femur, by splints or any restraining bandage; and, he adds, that he "cannot recollect an adult patient who did not halt for ever, after fracturing the neck of the thigh bone.

Some good remarks upon dislocations are given, and two cases are added, which are instructive in the mode of reduction. These we shall quote. "In a strong muscular man, whose thigh had been dislocated upwards and outwards, after fruitlessly trying other methods, the following process succeeded. He was laid prone upon a bed, a sheet was passed between his thighs, and held firmly by two assistants. I then knelt upon the pelvis in order to keep it steady, and resist its being raised up when the extension should be made. Three men then pulled at a towel fastened round the thigh above the knee, and drew it in such a direction as to carry the thigh upwards, that is, in relation to the trunk, backwards. I then rested my two hands on the head of the bone, and pushed it downwards and forwards with all my strength, and after a short exertion of our powers in this manner, I directed a gentleman who held the leg to twist the toes suddenly outwards, upon which the head rushed into the acetabalum with a loud noise.

"I tried the same and a variety of other methods in a very muscular middle-aged woman, unsuccessfully, within six hours after her accident. She took half a drachm of Dover's powder at bed time, the succeeding night and the next morning used the warm bath, and was well sweated for two hours before the intended time of repeating the taxis. She was laid upon the bed on the sound side. I then pressed my left hand against the head of the bone, one of my knees against its body, a little higher than the middle, and with the other hand, I drew her knee outwards. The leg was supported by an assistant, the knee bent to the right ancle. Three persons made steady the pelvis, by holding a sheet passed between the thighs, and three others made the extension. In this manner our strength was exerted for some time, and I plainly felt the head of the bone move, but the reduction was not compleated. We renewed our attempts in the same manner, except that a gentleman, who became one of the extenders, placed his foot firmly against the arch of the pubis (properly defended) and thereby both increased his power of extension, and at the same time rendered the pelvis more steady and fixed. The force being continued for some time, and my hands and knee being applied in the manner already described, I directed the assistant who supported the bent leg, suddenly to carry the internal ankle towards the other leg and to twist the toes outwards, and then the head slipped into the acetabalum."

The

The author adds some judicious observations on the reduction of the humerus, in which he recommends, above all other methods, for the surgeon to place a flattish ball or pad in the axilla, and over that a towel, which he ties over his own shoulders, and this must be so short that the operator must stoop considerably when it is fixed over his shoulder and the patient's axilla. The use of this contrivance is to apply to the head of the dislocated bone, the force of the surgeon exerted in raising his body, and to allow of his hands to be employed in directing the humerus and steadying the scapula. This is in fact the old fashioned implement of the kitchen towel and roller, which is known to every surgeon and bone-setter. The author notices the common objection to elevating the head of the bone on the supposition of the resistance opposed by the neck of the scapula, and thinks that the objection is not well founded, if the elevation be correctly made.

Some useful inferences may be drawn from these cases, of the importance of the operator himself taking as great a share in the manual part of the reduction as he well can, and rendering the efforts of the assistants as it were subordinate to his own. A full use should be made of the great purchase which the posture of kneeling or the resistance of the feet gives to a man in these circumstances. Persons who empirically practice bone-setting in the country, with no anatomical knowledge, nor any other advantage than a strong arm, and a kind of rough ingenuity in employing to the best effect their manual powers, though they often do irreparable mischief by their unregulated violence, frequently acquire great, and probably deserved reputation in this branch of surgery. Those, therefore, who have professional skill, should not hesitate to avail themselves of those methods, however rough and unsightly they may appear, which experience shews to possess great power, and which their anatomical knowledge enables them to regulate with safety.

---

*An Inquiry into the Efficacy of Oxygen in the Cure of Syphilis. To which are subjoined, a few general Observations on its Application in various other Disorders. By* C. PLATT, *Surgeon to the New Finsbury Dispensary, and Member of the Royal College of Surgeons, London.* 8vo. pp. 100. 1803.

As every Author must be supposed to understand his own views and motives, in publishing at least, as well as any other person, we consider it fair, in general, to give every one an opportunity of stating them in his own way. Mr. P. thus modestly introduces his pamphlet.

"The liberty I take to claim the indulgence of the public on a subject that hath been so ably discussed, may, perhaps, be considered rather arrogant, if not superfluous. In the prosecution of this design, however, I had in contemplation two objects; the one, to ascertain, by reference to practical experiments, the antidotal power of oxygen in syphilitic complaints; and the other, should my success not be commensurate with my expectation, to investigate gate the cause of such contrariety of sentiment among practitioners, who had the same object in view.

"About the termination of the fifteenth century, it is conjectured, the syphilitic disease was introduced into Europe from some of the new-discovered American islands. From that period to the present, much as we are indebted to the united abilities of so many intelligent writers who have favoured the world with their sentiments on this subject, still it is to be lamented, that a multiplicity of perplexing cases daily occurring, resist the common and generally adopted mode of treatment. Whether the want of more powerful remedies, or a more judicious administration of those already known, be the cause of this failure, is a difficulty often experienced, but not yet satisfactorily explained.

"No one, I am persuaded, will deny the advantage of frequently collecting the scattered though imperfect ideas that chance, or the labour and ingenuity of individuals, may have produced; and by occasionally forming a summary of these, it would be lamentable indeed, if some useful hint could not be derived, or some practical information inculcated.

"Our attention hath of late been very commendably excited by an ingenious mode of exhibiting oxygenous substances, as a remedy in eradicating syphilitic complaints. These saline substances unquestionably possess great activity, and are capable of producing some important changes in the animal economy; but whether the extent of the testimony already promulgated in their favour, entitles them to rank as specifics, so strenuously contended for by their advocates, can only be determined by an impartial investigation. Candour and truth compel me to observe, that the result of some experiments, carefully conducted, do not incline me to draw so favourable a conclusion. Having, from a variety of sources, acquired a strong opinion in favour of the antisyphilitic quality of the oxygenous substances, I neglected no opportunity that occurred, in the pursuit of so important a subject; and with what success, will be better determined after a perusal of the subsequent sheets."

The Author devotes the first twenty pages to a description of the principal symptoms of Lues, and a discussion of the much agitated question respecting the identity of the VIRUS in all the varieties of the disease. He considers the VIRUS the same in all; and imputes the variety of infection or symptoms, to the varied state or predisposition of the patient. Mr. P. next presents his Readers with a selection of cases, in which the oxygenated substances were employed without success.

This pamphlet appears to us to be written by a gentleman anxious to improve the scientific part of Surgery; and we are of opinion that it will be read by practitioners with approbation.

---

*Useful Hints to those who are afflicted with Ruptures; and on the Empirical Practices of the present Day.* By T. SHELDRAKE. 8vo. pp. 160. London, 1803.

EVERY attempt to alleviate human sufferings deserves attention; and although personal interest may be suspected of dictating some of the reasoning, yet the attempt should not, merely on that account, be entirely discouraged. Mr. S. thus explains his designs:

"The following observations were composed for the information of those persons, whose knowledge of ruptures was limited to the fact that they themselves were afflicted with the disease. Such may generally be divided into two classes, viz. those to whom the disease is an object of terror, and those who think it is of no consequence. The former torment themselves with needless apprehensions, and embitter their lives with perpetual anxiety to avoid evils that are created by their own imaginations; while the latter frequently betray themselves into imminent danger by their imprudent negligence.

"To re-assure the one, to caution the other, and to point out to both the only course that will place them in safety from the effects of this disease, are the objects which, it is hoped, may possibly be effected by this publication. It contains what may be called a popular account of the disease; i. e. such an account as professional men will allow to be true, and patients may understand, without possessing that information which professional men have on the subject.

"Besides investigating, in a general way, the principles on which trusses are applied to produce their effects, I have attempted to ascertain, to what kind of ruptures peculiar species of trusses may be best applied. This attempt must, from the nature of the subject, be imperfect; but, I trust, so much has been said as will demonstrate that no one kind of truss can be applicable to all ruptures indiscriminately; and this demonstration will prove the absurd pretensions and mischievous tendency of trusting on this subject to that description of persons, who having acquired the knack of making trusses in some particular manner, apply those trusses, indiscriminately, to every patient who comes in their way.

"Works like this generally fall into the hands of those who do not apply regularly for professional advice. To make it useful to this description of readers, I have inserted such information as can, without impropriety, be given in writing, with respect to the patient's management of himself, and the application of the truss. Much advice of this kind must be varied, according to circumstances, and adapted to individual cases; yet, as a kind of general system, if such a term may be used, I hope that what is contained on that head in the following pages will not be found entirely useless. In short, though it is possible that, from the various objects it embraces, few will take the trouble to read the whole, yet I am not without hopes that, whether the patient's object is to obtain obtain a just account of the various trusses that may be offered to his notice, or a rational idea of the principles on which trusses should be applied, to afford the best chance of being useful, he may find the following little work not entirely beneath his notice; and, on this account, though not without knowing it has many defects, and believing it may have many more than I actually know of, I cheerfully leave it to its fate."

